# Bacteriological quality and predictors of raw meat collected from municipal slaughterhouse and butcher shops in northeast, Ethiopia

**DOI:** 10.3389/fpubh.2024.1455881

**Published:** 2025-01-08

**Authors:** Leykun Berhanu, Adinew Gizeyatu, Masresha Abebe, Daniel Teshome, Mohammed Aragaw, Gete Berihun

**Affiliations:** ^1^Department of Environmental Health, College of Medicine and Health Sciences, Wollo University, Dessie, Ethiopia; ^2^Department of Biomedical Science, Asrat Woldeyes Health Science Campus, Debre Berhan University, Debre Berhan, Ethiopia; ^3^Department of Environmental Health, College of Medicine and Health Sciences, Debre Markos University, Debre Markos, Ethiopia

**Keywords:** slaughterhouse, meat, butcher shops, total viable count, Dessie City

## Abstract

**Background:**

Meat is a good source of protein in the human diet, and more than three-quarters of the world’s population consumes it. It is the most perishable food item since it has enough nutrients to enable microbial growth. In underdeveloped nations, animals are routinely slaughtered and sold in unsanitary conditions, compromising the bacteriological quality and safety of the meat received from the animals. To protect customers’ health from numerous foodborne diseases this study aimed to determine the microbial quality and predictors of meat along the meat value chain in Northeast, Ethiopia.

**Objective:**

To determine the bacteriological quality of meat and its predictors obtained from the Dessie City Administration’s municipal slaughterhouse and butcher shops.

**Materials and methods:**

A laboratory-based cross-sectional study design was used. In total 177 meat and 354 swab samples were collected. In addition, 177 meat handlers were randomly selected for knowledge, attitude, and practice assessment. All the samples were analyzed for the presence and counts of total viable count, total coliform count, fecal coliform count, and *S. aureus*. Multiple linear regression and student T-tests were used to analyze the data. Statistical significance was defined at a *p*-value of less than 0.05.

**Results:**

**T**he total viable count of meat samples collected from slaughterhouse and butcher shops were 5.17 ± 0.13 and 6.5 ± 0.87 log CFU/g, respectively. The overall mean total viable count of the meat samples was 5.8 ± 0.1 log CFU/g. Meat samples collected from butcher shops were more highly contaminated than those collected from slaughterhouse. Hand hygiene of meat handlers, the microbial quality of water, and the educational status of meat handlers are all significant predictors of the microbial quality of raw meat along the meat value chain.

**Conclusion and recommendation:**

The meat microbial quality is poor and deteriorates along the meat value chain. The hands of meat handlers, the microbial quality of water used to wash the hands of meat handlers, and the educational status of meat handlers significantly affect the microbial quality of raw meat along the meat value chain. Hence, measures should be taken to improve the personal hygiene status of meat handlers and the quality of water used to wash hands and meat contact surfaces.

## Introduction

Since meat is an excellent source of high-quality protein, fat, carbohydrates, vitamins, and minerals, as well as being tasty and easy to digest (1–3), it is consumed by 80% of the population globally (4, 5). However, it is an appropriate culture medium for many organisms due to its high moisture and nitrogen content (6–8) and high water activity level (9). Ethiopia has Africa’s largest cattle population, with 53.99 million cattle, 24 million sheep, and 18 million goats, contributing 40% of agricultural output and 15% of GDP. Cattle produce 0.331 million tons of meat annually (10). As a result, minced or raw beef is commonly used to prepare a popular traditional Ethiopian dish called “KITFO” in the local language, which is eaten raw or minimally cooked (2, 11). Meat can be contaminated at various stages of production, influenced by the animal’s condition at slaughter, temperature, and storage conditions (2). Animals should be slaughtered hygienically in licensed facilities that ensure sanitation and proper inspection for human use (12).

However, in underdeveloped nations including Ethiopia, animals are regularly slaughtered and sold in unsanitary conditions, compromising the microbiological quality and safety of the meat received from the animals (2, 13, 14). Meat products may also be contaminated with pathogenic microbes transmitted by meat handlers during the manufacturing, packing, and marketing (7, 15) by touching, breathing, coughing, or sneezing (7, 14). Contamination of raw meat also occurs from external sources during bleeding, handling, and processing via knives, tools, clothes, hands, and air (8). The bacteriological cleanliness of water used to clean hands and meat contact surfaces in slaughterhouses and butcher shops influences meat quality (13). In addition, meat may be spoiled because of its natural enzyme, microbial action, or other factors (2).

These contribute to the high contamination of meat. In meat sample aerobic bacterial count, total coliform, and *S. aureus* were reported to be 4.53, 3.97, and 3.88 log CFU/g, respectively (2). A similar study also found that the mean total viable bacteria, *Staphylococci*, Enterobacteriaceae, total coliform, fecal coliform, aerobic spore formers, yeasts, and molds of the butcher shops were 5.31, 4.24, 4.47, 4.79, 4.74, 3.77, and 5.0 log CFU/g, respectively (16). Another study also reported that the mean values of bacterial load of slaughterhouse meat, butcher shops, and street meat sales were 1.1*10^5^ CFU/g and 5.6*10^5^, respectively (17). Common foodborne bacterial pathogens in meat include *Salmonella*, *Staphylococcus aureus, E. coli, Campylobacter, Listeria, Clostridium, Yersinia,* and *Aeromonas* (8, 18). *Pseudomonas* species contribute to meat spoilage, causing off-odors, off-flavors, discoloration, and gas. Vibrio species are major causes of gastroenteritis, wound infections, and septicemia (8, 19). As a result, death costs billions of dollars in medical and societal expenditures in poor countries. Non-typhoidal *Salmonella* serotypes cause over 1.4 million cases, while pathogenic *E. coli,* cause 270,000 cases (8).

The study on the bacteriological quality of raw meat from municipal slaughterhouses and butcher shops in Northeast Ethiopia is important to assess food safety and public health risks associated with meat consumption. By identifying prevalent bacterial pathogens and their levels, the research provides crucial data for improving meat hygiene practices, informing regulatory policies, and enhancing consumer awareness regarding foodborne illnesses. Ultimately, it aims to reduce health risks and promote safer meat products in the region.

Several studies have been conducted in Ethiopia to determine the bacteriological quality of meat. However, none of them investigated the impact of meat handlers’ Knowledge, Attitude, and Practice (KAP), the bacteriological quality of water used to wash hands and meat contact surfaces, and the cleanliness of meat contact surfaces on the bacteriological quality of meat produced and sold in the study area. As a result, this study is crucial in determining the bacteriological quality and predictors of meat obtained from municipal slaughterhouses and butcher shops in Dessie, City administration.

## Materials and methods

### Study area

**Figure 1:**
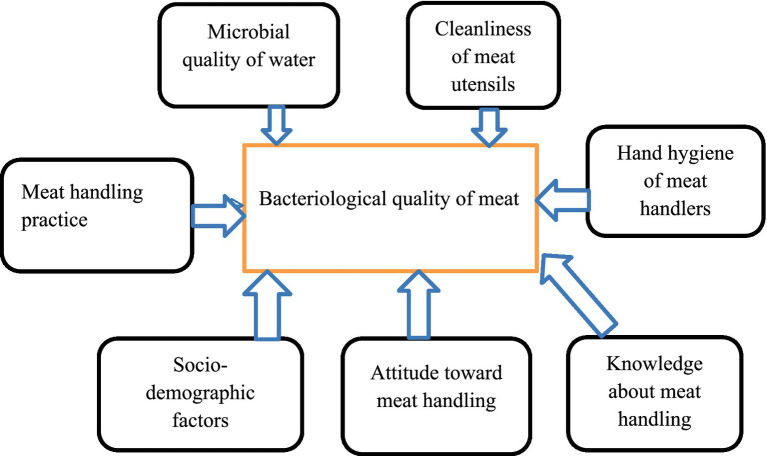
Conceptual framework of the study showing the relationship between dependent and independent variables.

The study was carried out in municipal slaughterhouse and butcher shops of the Dessie City administration. This city is located on the Addis Ababa-Mekele route in the Amhara Region’s South Wollo Administrative Zone, with latitude and longitude of 11′ 8°N 39′38°E with elevations of 2,470 and 2,550 meters, above sea level. The topography of Dessie is a highland type surrounded by ‘Tossa’ mountain. The annual maximum and minimum temperatures of Dessie are 23.7 °C and 9 °C , respectively. According to the Central Statistical Agency of Ethiopia, the population projection of the city was 212,436 comprising 105,011(49.4%) male and the remaining 107,425(50.6%) female (20). There are 18, 724 cattle, 22, 248 sheep, 2,572 goats, 1879 horses, 833 mules, 3,762 donkeys, and 37, 557 chickens in the city (21) (Figure 1).

### Study design and period

A laboratory-based cross-sectional study design was used to determine the bacteriological quality of meat and the various influencing factors. Data and samples were collected from September 2021 to January 2022.

### Population

### Source population

All meat slaughtering houses, butcher shops, knives, worker’s hands, cutting boards, and meat handlers working in slaughtering houses and butcher shops were the source population for this study.

### Study population

The study populations were selected from slaughtering houses, butcher shops, knives, worker’s hands, cutting boards, and meat handlers working in selected slaughtering houses and butcher shops.

### Eligibility criteria

The availability of meat slaughterhouses and butcher shops and the willingness of the meat handlers to be included in the study were the inclusion criteria for this study. In addition, meat handlers working for at least 6 months either in slaughterhouses or butcher shops were included in the study.

### Sample size and sampling technique

The number of meat samples required in this study was calculated based on the expected bacteriological contamination prevalence of the meat obtained from slaughterhouse and butcher shops, which was reported to be 6.5 and 5.8%, respectively (22). The sample size was calculated using the formula recommended by scholars (23).


$$n=\frac{1.96^{2}P\exp\;\;\left(1-P\exp\right)}{d^{2}}$$

There, n = required sample size, Pexp = expected prevalence (6.5% for slaughterhouse & 5.8% for butcher shops), and d = desired absolute precision (5%). Therefore, the required sample size for this study was 93 and 84 meat samples, from slaughterhouse and butcher shops, respectively. Hence, the total number of meat samples included in the study was 177. Similarly, 177 meat handlers (93 from slaughterhouse and 84 from butcher shops) were randomly selected for KAP assessment and hand hygiene exanimation. In addition, 177 water samples (93 from slaughterhouse and 84 from butcher shops) were collected. Moreover, 177 swab samples from knives (93 from slaughterhouse and 84 from butcher shops) were collected. From the meat cutting board, 177 surface swab samples were also collected (Table 1). Upon arrival to the slaughterhouse and butcher shops, owners and administrators was asked for their voluntariness. Simple random sampling technique was used to collected samples.

**Table 1:** Mean bacterial count of meat samples collected from slaughterhouse and butcher shops in Dessie City administration, Amhara region, Ethiopia.

Meat sample (n)	Mean bacterial count ± SD							
	Total viable count (Log CFU/g)		Total coliform count (Log CFU/g)		*Fecal coliform* count (Log CFU/g)		*Staphylococcus aureus* count (Log CFU/g)	
	Mean ± SD	*P*-value (95% CI)	Mean ± SD	*P*-value (95% CI)	Mean ± SD	*P*-value(95% CI)	Mean ± SD	*P*-value
Slaughterhouse (n = 93)	5.17 ± 0.13	≤0.001(−1.51)-(1.15)	3.93 ± 0.11	≤0.001(−0.14)-(−0.08)	3.61 ± 0.12	≤0.001(−0.19)-(−0.04)	3.03 ± 0.17	≤0.001(−0.35)-(−0.25)
Butcher shop (n = 84)	6.5 ± 0.87		4.04 ± 0.10		3.68 ± 0.11		3.33 ± 0.17	
Total (N = 177)	5.8 ± 0.1		3.98 ± 0.12		3.64 ± 0.12		3.17 ± 0.23	

SD, standard deviation; *S. aurues*, *Staphylococcus aurus*; CI, confidence interval.

### Sample and data collection

#### Meat sample collection

A meat cut sample of each 250 grams was collected from slaughterhouses and butcher shops (8). The sample was taken from the different regions of the carcasses including the lateral surface of the flank and rump, the loin, and the proximal part of the neck area using sterile polyethylene zip bags (8, 24, 25). The samples were collected between 7:00 to 9:00 am, i.e., post-slaughter and during early afternoons, to minimize the microbial changes due to environmental temperatures and post-slaughter timings. Then the sample bags were placed into the cold box containing icepacks and transported to the laboratory of the Department of Environmental Health for analysis within four hours of collection (2).

#### Environmental sample collection

Using a sterile, moistened cotton swab, samples were collected from meat-contact surfaces (meat handler’s hand, knives, and cutting board) over three-month period (10, 25, 26). The swab stick head was wetted using a drop of phosphate-buffered normal saline and rubbed slowly on the defined area of 1 cm^2^ in two directions at right angles to each other for 20 s. The samples were placed in labeled sterile tubes and then into cold iceboxes. It was then transported to the Environmental Health Laboratory at Wollo University for analysis within four hours of collection (10, 27–29). Additionally, a 100 mL water sample was collected directly from water sources the meat handlers used to wash their hands and meat contact surfaces by using sterile glass bottles (27). Then the sample glass was labeled, placed in a cold box with an ice pack, and transported to the laboratory of the Environmental Health Department for analysis within four hours of collection (27, 30).

### Data collection method and tool

The knowledge, attitude, and practice of meat handlers were assessed using a close-ended structured questionnaire.

### Sample analysis

#### Meat sample analysis

Twenty-five grams of meat sample was mixed with 225 mL of 0.1% Buffered Peptone Water (Merck, Darmstadt) and homogenized for 2 min by using a vortex mixer (Seward Ltd., UK) (2). Then one mL suspension was added into the test tubes containing 9 mL sterilized peptone water to make the first serial dilution (10^–1^). The process was continuing until 10 ^−5^. From the appropriate dilution, 0.1 mL was taken and spread plated in triplicate onto the Plate Count Agar (Oxoid, UK), MacConkey agar (Oxoid, UK), Mannitol Salt Agar (Oxoid, UK) (3, 31) to isolate total viable count, total coliform, and *S. aureus* count, respectively. Plates with 30 to 300 bacterial colonies were enumerated after incubation at 37 °C for 24 h (32). Yellow colonies growing on Mannitol Salt Agar were identified as positive for *S. aureus* (31, 33).The pink to red colonies were taken and streaked onto the Eosin Methylene Blue agar and were incubated at 37 °C for 24 h. After incubation, a green metallic sheen on the plate was identified as *E. coli* (14, 27). Four to five colonies were inoculated into tubes containing 2% Brilliant Green Bile Broth (Oxoid, UK) as a confirmatory test for coliform bacteria. Gas production as indicated by the rising of Durham tubes after 48 h of incubation at 35°C was used to assess the presence of coliform bacteria in a sample (34). The presumptive *S. aureus* bacteria were morphologically characterized by examining cultural characteristics such as color and shape. Biochemical characterization of isolates was done by carrying out, catalase test, oxidase test, motility test, and coagulase test (35).

#### Environmental sample analysis

The swabbed meat contact surfaces (meat handler’s hand swab, knife, and cutting board) were soaked into test tubes containing 9 mL sterilized peptone water. Serial dilution was prepared by transferring one mL of suspension and adding it into the test tubes containing nine mL of sterilized peptone water. Then 0.1 mL from the appropriate dilution was taken and spread plated in triplicate onto the sterile Petri Dishes containing plate count agar (Oxoid, UK), MacConkey agar (Oxoid, UK), and Manittol salt agar (Oxoid, UK) to determine the total viable count, coliform bacteria, and *S. aureus,* respectively. Then, the plates were incubated at 37 °C for 24 h. The number of different colonies was counted as colony forming unity per mL of sample volume plated on the plate using the dilution factor. The mean bacterial count was reported in log CFU/cm^2^ or per swab. Water samples were also examined using the same procedure (10).

### Microbial load determination

The microbial load was determined using the formula indicated below.

N = n/(s*d), where,

N = total number of bacteria per gram of the sample, “n” = average number of bacterial colonies, “d” is dilution factor of the food sample, and “s” is the volume of sample plated on the Petri Dish (16).

### Variables of the study

#### Dependent variable

- Bacteriological quality of meat

#### Independent variables

- Socio-demographic variables
- Bacteriological quality of the water
- Knowledge of meat handlers about safe meat handling
- Attitude of meat handlers toward safe meat handling
- Meat handling practice
- Bacteriological quality of meat contact surfaces
- Hand hygiene status of meat handlers

### Operational definition

#### Bacteriological quality of meat

The presence of total viable count and coliform bacteria in meat according to the limit (36, 37). Based on this, the microbial quality of meat due to total viable count was considered satisfactory if the total viable count in the meat sample is less than 10^5^ CFU/g (<5 log CFU/g), borderline if the total viable counts in the meat sample range from 10^5^ to 10^7^ CFU/g [5 to 7 log CFU/g], and unsatisfactory if the total viable count in the meat sample exceeds 10^7^ CFU/g (>7 log CFU/g).

#### Knowledge of meat handlers

The scoring system used to assess the respondent’s knowledge is based on the literature. The questions had two possible answers; each correct solution carried 2 marks while the wrong one carried one mark. In the case of negatively quoted questions, reverse scoring was used. Respondents who scored less than or equal to 50% were categorized as having poor knowledge, categorized as average knowledge if they scored 51 to 69.9%, and categorized as having good knowledge if they scored 70% and above (38).

#### Attitude toward meat handling

The evaluation of the attitude of meat handlers also depends on the literature. The questions had five possible answers strongly agree, agree, neutral, disagree, and strongly disagree which carry 4, 3, 2, 1, and 0 marks, respectively. For negatively quoted questions, reverse scoring was used. Then the subjects were classified as having a good attitude if they scored 70% and above, named as having a fair attitude if they scored 51 to 69.9%, and poor if they scored less than or equal to 50% (38).

#### The practice of meat handlers

The criteria used to evaluate the practice of meat handlers were obtained from the literature. The questions had always, often, sometimes, rarely, and never responses which carry 4, 3, 2, 1, and 0 marks, respectively. For negatively quoted questions, reverse scoring was used. Respondents were defined as having good practice if their score was greater than or equal to 70%, fair if their score was 51 to 69.9%, and poor if their score was less than or equal to 50% (38).

#### Quality of water used to wash hands and meat utensils

Water used to wash hands of meat handlers and meat utensils is considered good quality if no fecal coliform is present in the 100 mL water sample and poor quality if fecal coliform is detected in the 100 mL water sample (39).

#### Cleanliness of meat utensils

Was considered as clean if the coliform bacteria in the swab sample were not more than 10 CFU/equipment otherwise considered unclean (40).

#### Hand hygiene of meat handlers

The hands of meat handlers were considered clean if the total bacterial count was less than 100 CFU/cm^2^ per hand (or 2 log CFU/ cm^2^) otherwise considered unclean.

### Data collection method and tool

To evaluate meat handling knowledge, attitudes, and practices, a structured questionnaire was prepared. The questionnaire had four sections. The first part offers socio-demographic information about meat handlers. The second section includes questions to measure respondents’ knowledge of safe meat handling. The third section contains questions designed to assess the attitude of meat handlers toward safe meat handling and the last section consists of a list of questions designed to assess the practice of meat handling. The questionnaire was prepared in English and translated into the local language Amharic. The data was collected using a face-to-face interview and observational checklist.

### Data quality assurance

Before the beginning of the actual data collection, the questionnaire was pre-tested on 5% of the study subjects from other town’s municipality slaughterhouse and butcher shops. Based on the findings obtained, the necessary amendment was made. The principal investigator gave two days of training for data and sample collectors about the objective of the study, data collection tools, ethical issues, and other considerations that have to be cleared. Close supervision was made during the actual data and sample collection. On each day of the data collection period, the questionnaire was checked for its completeness and internal consistency. Laboratory instruments and measurements were calibrated and standardized. Sample analysis was carried out in triplicate with its control. All Media and reagents were of analytical grade. Sample analysis was carried out inside the level II safety cabinet. All equipment and culture media were sterilized using an autoclave and the adequacy of the sterilization process was assured using a sterilization indicator.

### Data management and statistical analysis

The data was entered into EpiData version 4.1 and exported to Statistical Package for Social Science version 25 software for data cleaning and analysis. Microbial counts of meat and environmental samples were recorded each day of counting. The bacterial count was transformed to log values and reported as mean ± standard deviation. T-test was calculated to determine whether there is a significant difference between the bacteriological quality of meat collected from slaughterhouses and butcher shops. Pearson correlation was also calculated to determine the presence of linear relationship between the bacteriological quality of meat and the quality of water, meat contact surfaces, knowledge, attitude, and practice of meat handlers toward safe meat handling practice. Linear regression was also employed to examine the influence of KAP of meat handlers, quality of water, and cleanliness of meat contact surfaces on the bacteriological quality of meat collected from the slaughtering houses and butcher shops. A *p*-value of less than 0.05 was considered statistically significant.

### Ethical consideration

Permission letters to carry out the study were obtained from the Research and Community Service offices of the College of Medicine, Wollo University. A formal letter of cooperation was written to the Dessie City administration. Necessary permission for data collection was taken from the city administration. Before starting the interview, the data collector explained the purpose of the study to all the participants, and verbal consent was obtained from the study participants. The confidentiality of the data was maintained by avoiding possible identifiers such as the names of the study participants instead, the identification code number was used as a reference. Those meat handlers identified as having poor hand hygiene had received adequate advice and consultation.

## Results

### Microbial quality of meat samples collected from slaughterhouse and butcher shops

The mean Total Viable Count (TVC) of the meat sample collected from slaughterhouse and butcher shops were 5.17 ± 0.13 and 6.5 ± 0.87 log CFU/g, respectively. The overall mean TVC of the samples was 5.8 ± 0.1 log CFU/g. The T-test revealed that there was a statistically significant difference (*p* ≤ 0.001 for all cases) between the mean TVC, TC, FC, and *S. aureus* of meat samples collected from slaughterhouse and butcher shops, where the meat samples collected from the butcher shops were the highly contaminated than the samples collected from the slaughterhouse (Table 1).

### Microbial quality of water samples collected from slaughterhouse and butcher shops

The mean TVC of water samples collected from slaughterhouse and butcher shops were 4.04 ± 0.17 and 4.14 ± 0.16 log CFU/mL, respectively. The finding obtained from the T-test indicated that there was a statistically significant difference between the mean TVC and FC of water samples collected from slaughterhouse and butcher shops. However, the mean TC of the water sample did not show a significant difference between the water samples collected from the slaughterhouse and butcher shops (Table 2).

**Table 2:** Mean bacterial count of water samples collected from slaughterhouse and butcher shops in Dessie City administration, Amhara region, Ethiopia.

Water sample	Mean bacterial count ± SD					
	Total viable count (Log CFU/mL)		Total coliform count (Log CFU/mL)		*Fecal coliform* count (Log CFU/mL)	
	Mean ± SD	*P*-value (95% CI)	Mean ± SD	*P*-value (95% CI)	Mean ± SD	*P*-value (95% CI)
Slaughterhouse (*n* = 93)	4.04 ± 0.17	≤0.001 (−0.14)-(−0.05)	3.80 ± 0.22	0.077 (−0.11)-(−0.01)	3.53 ± 0.08	0.019 (−0.06)-(−0.01)
Butcher shop (*n* = 84)	4.14 ± 0.16		3.86 ± 0.17		3.85 ± 0.17	
Total (*N* = 177)	4.09 ± 0.17		3.83 ± 0.2		3.58 ± 0.09	

CI, confidence interval; SD, standard deviation.

### Microbial quality of meat contact surfaces collected from slaughterhouse and butcher

The mean TVC of the swab sample collected from slaughterhouse and butcher shops was 4.16 ± 0.16 and 4.19 ± 0.16 log CFU/cm^2^, respectively. The overall mean TVC, TC, and FC of the cutting knives were 4.16 ± 0.18, 3.97 ± 0.16, 3.56 ± 0.15, log CFU/cm^2^, respectively. There was no statistically significant difference between the mean TVC, TC, and FC of swab samples collected from slaughterhouse and butcher shops (Table 3).

**Table 3:** Mean bacterial count of meat cutting knives collected from slaughterhouse and butcher shops in Dessie City administration, Amhara region, Ethiopia.

Cutting knife	Mean bacterial count ± SD					
	Total viable count (Log CFU/cm^2^)		Total coliform count (Log CFU/cm^2^)		*Fecal coliform* count (Log CFU/cm^2^)	
	Mean ± SD	*P*-value (95% CI)	Mean ± SD	*P*-value (95% CI)	Mean ± SD	*P*-value (95% CI)
Slaughterhouse (*n* = 93)	4.16 ± 0.16	0.263(−0.07–0.02)	3.99 ± 0.15	0.130(−0.01–0.08)	3.56 ± 0.13	0.699(−0.04–0.05)
Butcher shop (*n* = 84)	4.19 ± 0.16		3.95 ± 0.18		3.55 ± 0.18	
Total (*N* = 177)	4.16 ± 0.18		3.97 ± 0.16		3.56 ± 0.15	

CI, confidence interval; SD, standard deviation.

### Hand hygiene status of meat handlers

The mean TVC of hand swab samples collected from slaughterhouse and butcher shops were 5.02 ± 0.25 and 4.13 ± 0.15 log CFU/cm^2^, respectively. The T-test revealed that the TVC showed a statistically significant difference between those meat handlers working in slaughterhouse and butcher shops (Table 4).

**Table 4:** Mean bacterial count of swab samples collected from slaughterhouse and butcher shops in Dessie City administration, Amhara region, Ethiopia.

Hand swab samples	Mean bacterial count ± SD					
	Total viable count (log CFU/cm^2^)		Total coliform count (Log CFU/cm^2^)		Fecal coliform count (Log CFU/cm^2^)	
	Mean ± SD	*P*-value (95% CI)	Mean ± SD	*P*-value (95% CI)	Mean ± SD	*P*-value (95% CI)
slaughterhouse (*n* = 93)	5.02 ± 0.25	≤0.001(0.82–0.95)	3.77 ± 0.25	≤0.001(−0.19-(−0.07))	3.39 ± 0.23	≤0.001(−0.17-(−0.05))
Butcher shop (*n* = 84)	4.13 ± 0.15		3.90 ± 0.11		3.5 ± 0.13	
Total (*N* = 177)	4.60 ± 0.50		3.83 ± 0.21		3.44 ± 0.20	

CI, confidence interval; SD, standard deviation.

### Knowledge, attitude, and practices of meat handlers

#### Socio-demographic characteristics of meat handlers

All 177 study participants have finished the interview, resulting in a 100% response rate. Two-thirds of the respondents, 68 (38.4%), were between the ages of 25 and 34, and more than two-thirds, 122 (68.9%), were men. About one-third, 54 (30.4%), of the respondents said that they had finished secondary school, while less than one-tenth, 11 (6.2%), could not read and write (Table 5).

**Table 5:** Socio-demographic characteristics of meat handlers working in slaughterhouse and butcher shops in Dessie City administration, Amhara region, Ethiopia.

Sr.no	List of variables	Responses	Frequency	Percent
1.	Sex	Male	122	68.9
		Female	55	31.1
		Total	177	100
2.	Age	18–24	42	23.7
		25–34	68	38.4
		35–44	45	25.4
		≥45	22	12.4
		Total	177	100
3.	Educational status	Cannot read and write	11	6.2
		Can read and write	31	17.5
		Primary school (1–8 grades)	52	29.4
		Secondary school (9–12 grades)	54	30.5
		College diploma and above	29	16.4
		Total	177	100
4.	Marital status	Single	62	35.0
		Married	60	33.9
		Divorced	43	24.3
		Widowed	12	6.8
		Total	177	100
5.	Religion	Orthodox	112	63.3
		Muslim	41	23.2
		Protestant	24	13.6
		Total	177	100
6.	Family size	Less than or equal to five	120	67.8
		Greater than five	57	32.2
		Total	177	100

### Knowledge, attitude, and practice score of meat handlers

The mean knowledge, attitude, and practice scores of the respondents working in slaughterhouse were 70.57% ± 19.28, 64.74% ± 11.45, and 61.57% ± 12.76, respectively. The mean knowledge, attitude, and practice scores of the respondents working in butcher shops were 70.59% ± 20.94, 64.90% ± 9.56, and 56.78% ± 15.73, respectively. The T-test indicated no statistically significant difference in the knowledge and attitude scores of the meat handlers working in slaughterhouse and butcher shops. However, the mean attitude score showed a statistically significant difference (Table 6).

**Table 6:** Mean Knowledge, attitude, and practice score of the respondents working in slaughterhouse and butcher shops of Dessie city administration.

Workplace	Mean Knowledge score ± SD	*P*-value	Mean attitude score ± SD	*P*-value	Mean practice scores	*P*-value
slaughterhouse (*n* = 93)	70.57 ± 19.28	0.505(−7.98–3.95)	64.74 ± 11.45	0.925(−3.30–3.00)	61.57 ± 12.76	0.027(0.56–9.02)
Butcher shops (*n* = 84)	70.59 ± 20.94		64.90 ± 9.56		56.78 ± 15.73	
Overall score (*n* = 117)	71.5 ± 20.05		64.8 ± 10.57		59.3 ± 14.4	

SD, Standard deviation.

### Level of knowledge, attitude, and practices of meat handlers

Among 177 meat handlers included in the study, half, 88(49.7%), third 58(32.8%), and two fifth 42(23.7%) of them had a good level of knowledge, attitude, and practices, respectively. Among meat handlers working in slaughterhouse more than half, 44(47.3%) of them had a good level of knowledge about meat handling. Regarding the meat handling practices, among those meat handlers working in slaughterhouse about one-fourth, 25(26.7%) of them had good meat handling practices while only about one-tenth, 13(14.0%) of the meat handlers had good levels of practices (Table 7).

**Table 7:** Level of knowledge, attitude, and practices of meat handlers working in slaughterhouse and butcher shops of Dessie City administration, Northeast Ethiopia, Amhara Region.

Meat handlers	Category of knowledge			Category of attitude			Category of practice		
	Good *n* (%)	Fair *n* (%)	Poor *n* (%)	Good *n* (%)	Fair *n* (%)	Poor *n* (%)	Good *n* (%)	Fair *n* (%)	Practice *n* (%)
slaughterhouse (*n* = 93)	44 (47.3)	40 (43.0)	9 (9.7)	34 (36.6)	51 (54.8)	8 (8.6)	25 (26.9)	55 (59.1)	13 (14.0)
Butcher shops (*n* = 84)	44 (52.4)	30 (35.7)	10 (11.9)	24 (28.6)	56 (66.7)	4 (4.8)	17 (20.2)	42 (50)	25 (29.8)
Total (*n* = 177)	88 (49.7)	70 (39.5)	19 (10.7)	58 (32.8)	107 (60.5)	12 (6.8)	42 (23.7)	97 (54.7)	38 (21.5)

### Predictors of meat quality

After checking the assumption for linear regression all the variables sex, age, educational status, marital status, religion, knowledge about meat handling, attitude toward meat handling, practice of meat handling, water quality, hand hygiene, and microbial quality of meat contact surfaces were entered into the multiple linear regression model.

The multiple linear regression models indicated that microbial load of hand hygiene, water quality, and educational status of the meat handlers were the three major explanatory variables that have a great influence on the microbial quality of meat in the study area.

As the number of microbial loads on the hands of meat handlers increased by one unit, the meat microbial quality decreased by 1.13. It clearly showed that improving the hand hygiene status of meat handlers has contributed to improving the microbial quality of meat. In addition, as the microbial load of water used to wash handlers or process meat increased by one unit, then the microbial quality of meat reduced by 0.778. Moreover, as the educational status of the respondents increased by one unit, the microbiological quality of meat increased by 0.1. It indicated that increasing the educational status of the respondents plays a great role in improving the microbial quality of the meat in slaughterhouse and butcher shops (Table 8).

**Table 8:** Predictors of meat quality along the meat value chain in Dessie City administration, Northeast Ethiopia.

Model		Unstandardized coefficients		Standardized coefficients	*P*-value	95.0% Confidence Interval for B	
		B	Std. Error	Beta		Lower bound	Upper bound
1	(Constant)	11.552	2.196		0.000	7.215	15.889
	Sex of the respondent	−0.134	0.117	−0.068	0.254	−0.364	0.097
	Age of the respondent	0.003	0.055	0.003	0.956	−0.106	0.112
	Educational status of the meat handlers	0.100	0.049	0.124	0.044*	0.003	0.197
	Marital status	−0.115	0.058	−0.118	0.050	−0.231	0.000
	Religion of the respondent	0.079	0.077	0.063	0.308	−0.073	0.231
	Attitude score (100%)	0.010	0.005	0.120	0.054	0.000	0.021
	Practice score (100%)	−0.003	0.004	−0.044	0.481	−0.010	0.005
	Water quality	−0.778	0.355	−0.129	0.030*	−1.479	−0.076
	Meat contact surface microbial quality	0.525	0.377	0.083	0.165	−0.219	1.269
	Knowledge score	−0.003	0.003	−0.071	0.243	−0.009	0.002
	Hand swab samples	−1.130	0.115	−0.598	≤0.001*	−1.358	−0.902

*Indicates the presence of statistical association.

## Discussion

Consuming raw meat is widespread and linked to cultural customs in Ethiopia. According to a recent study, regularly eating raw meat exposed almost 60% of the respondents to the risk of zoonotic infections (41). As a result, it is critical to maintain the microbiological safety of meat and prevent foodborne illnesses in butcher shops and slaughterhouse by implementing stringent hygiene protocols, providing education and media campaigns, encouraging changes in consumer behavior about meat origin, and implementing adequate sanitation measures (41, 42). Determining the microbiological quality and predictor of raw meat obtained from butcher shops and slaughterhouse is therefore the main goal of this study. TVC testing helps measure bacterial presence and assesses hygiene in meat processing. High TVC in meat can impact marketability and consumer acceptability (43).

The average viable count of the meat samples taken from the slaughterhouse in this investigation was 5.17 ± 0.13 log CFU/g. Higher results were reported from Egypt 6.45 log CFU/g (7), in Debre Birhan 5.31 log CFU/g (16), in Northern India 6.0 log CFU/g (32), in Pakistan 10 log CFU/g (44), and in Uganda 8.21 log CFU/g (45) was reported. A high total viable count in meat samples indicates inadequate sanitation and hygiene conditions in the slaughterhouse, which could result in foodborne illnesses and even mortality from pathogens that enter the body through ingestion (46). In the contrary, lower results were also reported in various studies including; in Assosa 4.03 log CFU/g (3), in Kenya 3.35 log CFU/g (25), in Bishoftu 2.4 log CFU/g (46). The possible reason for the difference might be due to the difference in study setting, weather condition, and the difference in laboratory method.

The TVC of meat samples obtained from butcher shops was 6.5 ± 0.87 log CFU/g in this investigation. The mean total viable count of meat samples obtained from butcher shops was reported to be 4.53 log CFU/g in Bahir Dar (2), 5.75 log CFU/g in Mekelle (17), and 5.47 log CFU/g in Debre Birhan (47). These results represented the lower viable count. The disparity in sample time, hand cleanliness habits, and variations in ambient temperature could contribute to this discrepancy (47). A high level of TVC can result from meat handling, processing procedures, storage conditions, and hygiene practices. Variations may also arise from differences in sampling plans, sample sizes, and laboratory methods used for microbiological analysis (16). In contrast, higher viable count values were found in Uganda (3), Pakistan (17), and the area around Addis Ababa, where the mean viable count was reported to be 8.28, 8.09, and 9.81 log CFU/g, respectively. Concerns arise from an abnormally high TVC in a meat sample, as it can lead to reduced shelf life, sanitary issues, regulatory noncompliance, and negative consumer perceptions. This highlights the importance of maintaining hygiene standards, using proper production processes, and conducting regular microbiological testing to ensure meat safety and quality.

Furthermore, a high total viable count in meat samples taken from butcher shops indicates improper handling and inadequate cleanliness, which can result in foodborne infections such as *E. coli*, salmonellosis, and campylobacteriosis. To avoid contamination and reduce the risk of foodborne illness, meat should be handled and processed hygienically (48). Spoilage microorganisms such as mold, yeast, and bacteria are frequently associated with a high total viable population. These microbes can alter the color, texture, flavor, and odor of meat, lowering its shelf life and quality. Meat with a high TVC concentration is more likely to degrade quickly, reducing its marketability and consumer acceptance (49). The study found that the average fecal coliform count in meat samples collected from butcher shops and slaughterhouse was 3.61 ± 0.12 and 3.03 ± 0.17 log CFU/g, respectively. These results fall into the category of borderline microbial quality, meaning that the microbial counts in the meat sample are either slightly above or close to the acceptable limits specified by regulatory standards (50).

The butcher shop and slaughterhouse swab samples used in this study had a mean total viable count of 4.19 ± 0.16 and 4.16 ± 0.16 log CFU/ cm^2^, respectively. In Pakistan, where the mean total viable count was 10.2 log CFU/cm^2^, a higher total viable count was recorded (26). High viable counts and pathogens on meat-processing knives, walls, and floors reflect environmental hygiene. Contamination can occur from bacteria on surfaces and equipment, significantly affecting meat quality. When meat contacts knives with high total viable counts, cross-contamination happens, allowing harmful bacteria to migrate into the meat, jeopardizing its safety and quality (45). Pathogenic microorganisms such as *Salmonella*, *E. coli, Campylobacter*, and *L. monocytogenes* may be present in the knives. Contamination from microbes on cutting knives can increase the risk of foodborne illnesses. High TVC on knives can transfer bacteria to meat, altering its color, texture, and flavor, making it unfit for consumption. This may also violate food safety regulations. To mitigate these risks, it is essential to follow proper cleanliness procedures, regularly clean and sanitize knives, and maintain good production practices in handling, storing, and maintaining equipment to ensure meat safety (50).

Furthermore, Berhanu and his colleagues (27) and Mansouri-Najand and colleagues (51) provided additional evidence for this conclusion by showing that food handlers’ educational attainment has a major impact on the caliber of the food they handle. Their findings suggest that a greater awareness of food safety procedures, appropriate hygiene, and efficient handling methods are frequently linked to food handlers’ higher educational attainment. According to this association, funding food handler education initiatives may produce better food safety and quality results, lowering the risk of foodborne diseases and boosting consumer trust in food items.

Using water of low microbiological quality for washing or chilling meat can introduce pathogenic or spoilage bacteria, raising the risk of foodborne illness and increasing microbial load. This water can cause cross-contamination, spreading harmful germs to both meat and processing surfaces, which further jeopardizes food safety by perpetuating contamination cycles (52). Water with high microbial populations or specific spoilage bacteria can accelerate meat deterioration, reducing its shelf life and consumer appeal due to changes in taste, odor, and appearance. Poor-quality water also fails to adequately clean and sterilize surfaces, utensils, and equipment, increasing the risk of contamination in later processing steps by allowing pathogenic or spoilage bacteria to survive and thrive (53).

This study highlights food safety and hygiene practices related to meat consumption, addressing a significant public health issue. It evaluates the microbiological quality of meat and identifies contamination levels using hygiene indicators and pathogens. A key strength is its focus on factors often overlooked in food safety studies, such as water quality for cleaning, meat handlers’ knowledge, and the condition of meat contact surfaces. By examining local slaughterhouses and butcher shops, the study enhances understanding of community practices, guiding initiatives to improve hygiene and reduce contamination risks. It provides a foundation for future research to monitor changes in meat quality and can inform legislators and health officials to improve regulations for meat handling and processing.

The study has limitations, as hygiene indicator bacteria do not identify all pathogens in raw meat and do not cover all harmful bacteria that may pose risks to consumers. Additionally, not all factors affecting meat quality, such as feed, transportation, and animal health, were examined. The authors recommend that future research focus on foodborne pathogens in raw meat and consider transportation conditions and animal health as potential contamination sources in the supply chain. Using cultural method in this research is time consuming since microorganism requires several times to grow. There is also challenge of culturing organisms that cannot be cultured leads to an underappreciation of microbial diversity. Lastly, cultural techniques can be demanding in terms of labor, necessitating a substantial level of expertise and effort, highlighting the need to incorporate molecular methods for a more thorough understanding of microbial communities.

## Conclusion and recommendation

The present study revealed that the meat microbial quality is poor. The hands of meat handlers, the microbial quality of water used to wash the hands of meat handlers, and the educational status of meat handlers significantly affect the microbial quality of raw meat in slaughterhouses and butcher shops. In light of the findings, steps should be taken to enforce strict hygiene protocols for meat handlers, such as frequent hand washing with potable water and the use of sanitizers. Training sessions should also stress the importance of maintaining personal hygiene. Develop and implement comprehensive training programs for meat handlers that highlight proper handling techniques, food safety protocols, and hygiene in slaughterhouses and butcher shops play a key role to enhance the microbial quality of the meat.

## Data Availability

The raw data supporting the conclusions of this article will be made available by the authors, without undue reservation.
