# Supplementary material for: Bacteriological quality and predictors of raw meat collected from municipal slaughterhouse and butcher shops in northeast, Ethiopia
Source: Front Public Health. 2025 Jan 8;12:1455881. doi: 10.3389/fpubh.2024.1455881 (PMC11751221; doi:10.3389/fpubh.2024.1455881)
Supplement: Supplementary file 1 [file Table_1.DOCX]

# Annex I: Questionnaire designed to assess the knowledge, attitude, and practice of meat handlers

**Information sheet and Consent form**

My name is________ lecturer and researcher at Department of Environmental Health, College of Medicine and Health Science, Wollo University. I am conducting a research on” Bacteriological Quality of Meat and Its Predictors Collected from Municipal Slaughter houses and Butcher shops in Dessie City Administration” The main aim of the study is to determine the bacteriological quality of meat and its predictors collected from municipal slaughter house and butcher shops in Dessie City Administration. The questionnaire will consist of four parts including; socio demographic characteristic of the respondents, knowledge about meat handling, attitude towards meat handling and meat handling practices.

Your answers will be recorded on a survey questionnaire. No personal identifiers will be recorded to the interview. All the data obtained will be kept strictly confidential by using only code numbers. Your participation in the study is upon purely voluntary basis. Your information is vital, without which the realization of the research project would be impractical. Moreover, what we learn from this study will be used to generate information necessary to design meat-handling policy.

The interview will take 15-20 minute. During the interview period, if you feel inconvenient, you can interrupt and clarify inconvenience, appoint to other time or even withdraw any time after you get involved in the study.

If yes, go on.

*Thank you for your cooperation!*

**Part One: Socio demographic characteristics of the respondents**

| **Sr.no** | **List of variables** | **Response** |
| --- | --- | --- |
| 1.1 | Sex of the respondent | Male__ _Female____ |
| 1.2 | Age of the respondent | ________ |
| 1.3 | Educational status of the | A. No education C. 1-4 E.9-10 G. >12  B. Read and write D. 5-8 F.11-12 |
| 1.4 | Marital status | 1. Single C. Widowed 2. Married D. Divorced |
| 1.5 | Religion of the respondent | 1. Muslim C. Protestant 2. Orthodox D. Other specify |
| 1.6 | Ethnicity | 1. Amhara B. Tigray E. Argoba 2. Oromo D. Afar F. None |
| 1.7 | Total family size | __________ |

**Part Two: Questions designed to assess the knowledge of meat handlers**

| **Sr.no** | **List of questions** | | | **Response** | | | | Remark | | | |
| --- | --- | --- | --- | --- | --- | --- | --- | --- | --- | --- | --- |
|  |  |  |  | Yes | | No | |  |  |  |  |
| 2.1 | Wearing glove during meat handling is one part of personal hygiene | | |  | |  | |  | | | |
| 2.2 | Wearing mask during meat handling is one part of personal hygiene | | |  | |  | |  | | | |
| 2.3 | Covering hair during meat handling is one part of my job responsibility | | |  | |  | |  | | | |
| 2.4 | Washing hands regularly before work is one part of personal hygiene | | |  | |  | |  | | | |
| 2.5 | Washing hands regularly after touching nose, doors, tables or any inanimate object is one part of personal hygiene | | |  | |  | |  | | | |
| 2.6 | Washing hands with only water is clean enough to prevent food borne illness | | |  | |  | |  | | | |
| 2.7 | Washing hands with hot water and soap is required to prevent food borne illness | | |  | |  | |  | | | |
| 2.8 | Meat handlers should wash hands after touching any part of his/her body. | | |  | |  | |  | | | |
| 2.9 | Washing hands with soap and water after touching raw meat is enough to prevent contamination | | |  | |  | |  | | | |
| 2.10 | Meat handlers should wash hands before and after using gloves | | |  | |  | |  | | | |
| 2.11 | Hands are most sources of bacteria and microbes | | |  | |  | |  | | | |
| 2.12 | When coughing or sneezing covering mouth and nose prevents spread of infection | | |  | |  | |  | | | |
| 2.13 | Long hair could transmit microbes to food when head is not covered while handling food | | |  | |  | |  | | | |
| 2.14 | Maintaining personal hygiene is important to control food borne disease | | |  | |  | |  | | | |
| 2.15 | Pets in food premises can contaminate meat | | |  | |  | |  | | | |
| 2.16 | Foodborne pathogens can be seen by the eye | | |  | |  | |  | | | |
| 2.17 | Meat serving cups can transfer diseases causing agent to the consumers | | |  | |  | |  | | | |
| 2.18 | Washing meat contact equipment with only hot water is enough | | |  | |  | |  | | | |
| 2.19 | Washing serving cups with only hot water is enough | | |  | |  | |  | | | |
| 2.20 | Washing meat contact utensils with hot water and soap provide good protection against the transfer of harmful pathogen to the consumers | | |  | |  | |  | | | |
| 2.21 | Meat preparation must be done using clean equipment. | | |  | |  | |  | | | |
| 2.22 | The cooking equipment should be washed immediately after use | | |  | |  | |  | | | |
| 2.23 | Employees should avoid touching their hair after washing hands | | |  | |  | |  | | | |
| 2.24 | Wearing bracelet, rings and other adornments is not allowed in the work place | | |  | |  | |  | | | |
| 2.25 | Meat handlers cannot have long nails and make coloring it | | |  | |  | |  | | | |
| 2.26 | When meat handlers have wound on hands use plaster and do not perform normal job | | |  | |  | |  | | | |
| 2.27 | Contamination is the transfer of harmful microorganisms to food from other foods or non-food-contact surfaces | | |  | |  | |  | | | |
| 2.28 | If I want to draw water from storage container, I will use dipping method | | |  | |  | |  | | | |
| 2.29 | Cleaning equipment after work can reduce cross contamination | | |  | |  | |  | | | |
| 2.30 | Diarrhea is a disease occurred when people eat unclean food | | |  | |  | |  | | | |
| 2.31 | Diarrhea can be transmitted from people to others | | |  | |  | |  | | | |
| 2.32 | Food storage time is one of the important factors to control growth of bacteria | | |  | |  | |  | | | |
| 2.33 | Refrigeration temperature is one of the important factors to control growth of bacteria | | |  | |  | |  | | | |
| 2.34 | Chilling process cannot kill all of the bacteria | | |  | |  | |  | | | |
| 2.35 | Cooking can destroy any form of bacteria | | |  | |  | |  | | | |
| **Part three:** Questions designed to assess the attitude of meat handlers | | | | | | | | | | | |
| **S/n** | **List of questions** | | **0** | | **1** | | **2** | | **3** | | **4** |
| 3.1 | Safe meat handling is an important part of my job responsibility | |  | |  | |  | |  | |  |
| 3.2 | I will change my food handling behavior, if I know it is incorrect | |  | |  | |  | |  | |  |
| 3.3 | I believe food safety knowledge will benefit to my personal life | |  | |  | |  | |  | |  |
| 3.4 | I believe food safety knowledge will benefit to consumers | |  | |  | |  | |  | |  |
| 3.5 | I would attend a cooking or service competition to improve my professional knowledge | |  | |  | |  | |  | |  |
| 3.6 | Consumers are responsible to prevent food poisoning | |  | |  | |  | |  | |  |
| 3.7 | Government is responsible to prevent food poisoning | |  | |  | |  | |  | |  |
| 3.8 | We should not rub our hands on face, hair, etc. while working | |  | |  | |  | |  | |  |
| 3.9 | I use the same towel to clean many places. | |  | |  | |  | |  | |  |
| 3.10 | I am willing to learn about the basics of food hygiene and safety | |  | |  | |  | |  | |  |
| 3.11 | Producing safe food is more important than tasty food | |  | |  | |  | |  | |  |
| 3.12 | I believe good personal hygiene can prevent food-borne illness | |  | |  | |  | |  | |  |
| 3.13 | Washing hand before handling meat reduces risk of food poisoning | |  | |  | |  | |  | |  |
| 3.14 | Worker should make sure that their nails are short and clean | |  | |  | |  | |  | |  |
| 3.15 | I encourage food handlers to get vaccinated | |  | |  | |  | |  | |  |
| 3.16 | Touching raw or cooked food without gloves lead to contamination and cause food poisoning | |  | |  | |  | |  | |  |
| 3.17 | Cover your mouth while coughing to prevent spread of infection | |  | |  | |  | |  | |  |
| 3.18 | Do you prefer wearing ring or watch inside while you handle meat? | |  | |  | |  | |  | |  |
| 3.19 | Cutting hair every 4-5 weeks or trimming is healthy | |  | |  | |  | |  | |  |
| 3.20 | Not wearing rings and watch during food handling | |  | |  | |  | |  | |  |
| 3.21 | Workers with abrasion or cuts on fingers and hands can handle food without gloves | |  | |  | |  | |  | |  |
| 3.22 | I can handle food even I get sick, fever or catch cold | |  | |  | |  | |  | |  |
| 3.23 | I can handle meat when I get diarrhea | |  | |  | |  | |  | |  |
| 3.24 | Using mask during handling is important in reducing risk of food contamination | |  | |  | |  | |  | |  |
| 3.25 | Using cap or hair cover is important in reducing risk of food contamination | |  | |  | |  | |  | |  |
| 3.26 | Using gloves during meat handling is important in reducing risk of food contamination | |  | |  | |  | |  | |  |
| 3.27 | The use of adornments, such as earrings, rings and watches, during meat handling cannot cause food contamination | |  | |  | |  | |  | |  |
| 3.28 | It is necessary to check temperature settings of chillers/refrigerators | |  | |  | |  | |  | |  |
| 3.29 | You can tell if meat is safe by looking at it | |  | |  | |  | |  | |  |
| 3.30 | Raw and processed meat should be stored separately | |  | |  | |  | |  | |  |
| **KEY: 0 = Strongly disagree, 1 = Disagree, 2 = Neutral, 3 = Agree 4 = Strongly agree** | | | | | | | | | | | |
| **Part Four: Questions developed to assess the meat handling practice** | | | | | | | | | | | |
| **S. n** | **List of questions** | **Response** | | | | | | | | | |
|  |  | **0** | | **1** | | **2** | | **3** | | **4** | |
| 4.1 | Do you wash your hands before processing meat? |  | |  | |  | |  | |  | |
| 4.2 | Do you use detergent to wash your hands? |  | |  | |  | |  | |  | |
| 4.3 | Do you wash meat contact surfaces (knives, cutting board, tables….etc)? |  | |  | |  | |  | |  | |
| 4.4 | Do you use detergent to wash meat utensils? |  | |  | |  | |  | |  | |
| 4.5 | Do you keep your nails short during meat handling? |  | |  | |  | |  | |  | |
| 4.6 | Do you remove all adornments before starting meat-handling activities? |  | |  | |  | |  | |  | |
| 4.7 | Do you handle meat at work when you have diarrhea? |  | |  | |  | |  | |  | |
| 4.8 | Do you wear clean cloth when you are in work place? |  | |  | |  | |  | |  | |
| 4.9 | Do you wear jewelry, rings and watches? |  | |  | |  | |  | |  | |
| 4.10 | Do you smoke cigarette when you handle meat? |  | |  | |  | |  | |  | |
| 4.11 | Do you wash your hands after touching money? |  | |  | |  | |  | |  | |
| 4.12 | Do you wash your hands after cleaning table? |  | |  | |  | |  | |  | |
| 4.13 | Do you wash cooking equipment immediately after use |  | |  | |  | |  | |  | |
| 4.14 | Do you wash your hands for at least 20 seconds? |  | |  | |  | |  | |  | |
| 4.15 | Do you cover food properly to prevent contamination? |  | |  | |  | |  | |  | |
| 4.16 | Do you handle meat at work when you have abrasions or cuts on your hands? |  | |  | |  | |  | |  | |
| 4.17 | Do you wash your hands after go to toilet? |  | |  | |  | |  | |  | |
| 4.18 | Do you use mask during handling meat? |  | |  | |  | |  | |  | |
| 4.19 | Do you use cap or hair cover during handling meat? |  | |  | |  | |  | |  | |
| 4.20 | Do you use gloves on both hands at work during handling meat? |  | |  | |  | |  | |  | |
| 4.21 | Do you take periodic physical examination? |  | |  | |  | |  | |  | |
| 4.22 | Do you sanitize your workplace after finishing your service? |  | |  | |  | |  | |  | |
| 4.23 | Do you thaw food at room temperature (outside the fridge)? |  | |  | |  | |  | |  | |
| **Key: 0 = never 1 = Rarely 2 = Sometimes 3 = Often 4 = Always** | | | | | | | | | | | |
